# Supplementary material for: Quantification of the capacity of vibrio fischeri to establish symbiosis with Euprymna scolopes
Source: PLoS One. 2023 Jul 13;18(7):e0287519. doi: 10.1371/journal.pone.0287519 (PMC10343157; doi:10.1371/journal.pone.0287519)
Supplement: S1 File — (PDF) [file pone.0287519.s001.pdf]

Symbiotic Dose-50 (SD50) for Vibrio fischeri strain to colonize Euprymna scolopes V.2

ard[1,2], ejg[1,2], agc[1,2], Tim I Miyashiro[1,2]

[1]Department of Biochemistry and Molecular Biology, The Pennsylvania State University, University Park, PA, USA;
[2]The One Health Microbiome Center, Huck Institutes of the Life Sciences, The Pennsylvania State University, University Park, PA, USA

Tim I Miyashiro

VERSION 2

JUL 01, 2023

OPEN ACCESS

DOI:
dx.doi.org/10.17504/protocols.io.yxmvm2155g3p/v2

Protocol Citation: ard, ejg, agc, Tim I Miyashiro 2023. Symbiotic Dose-50 (SD50) for Vibrio fischeri strain to colonize Euprymna scolopes. **protocols.io** https://dx.doi.org/10.17504/protocols.io.yxmvm2155g3p/v2Version created by Tim I Miyashiro

License: This is an open access protocol distributed under the terms of the Creative Commons Attribution License, which permits unrestricted use, distribution, and reproduction in any medium, provided the original author and source are credited

Protocol status: Working
We use this protocol and it's working

Created: Jun 30, 2023

Last Modified: Jul 01, 2023

PROTOCOL integer ID: 84312

ABSTRACT

This protocol details symbiotic dose-50 (SD_{50}) for *Vibrio fischeri* strain to colonize *Euprymna scolopes*.

MATERIALS

Materials needed:

1. Culture tubes
2. LBS medium [1% (w/v) tryptone, 0.5% (w/v) yeast extract, 2% (w/v) NaCl, 50 mM Tris–HCl (pH 7.5)], with
   1.5% w/v agar for solid medium
3. Shaking incubator at 28°C
4. Spectrophotometer and cuvettes
5. Plastic Tumblers, e.g., Fineline Mfr. #409-CL Savvi Serve 9 oz. Clear Hard Plastic
6. Freshly hatched *E. scolopes* squid
7. Transfer pipets, e.g., Fisherbrand Disposable Graduated Transfer Pipettes Catalog No. 13-711-9AM
8. Filter-sterilized seawater (FSSW): Instant Ocean (Spectrum Brands, Blacksburg, VA) mixed according to instructions provided by manufacturer. Filter through 0.22-µm surfactant-free filter (Nalgene Rapid-Flow Sterile Disposable Filter Units with SFCA Membranes).
9. Microfuge tubes
10. 50-mL conical tubes
11. Vials, e.g., VWR Drosophila vials narrow #75813-162
12. Luminometer, e.g., GloMax 20/20 (Promega Corp., Madison, WI)

Keywords: Preparation of V. fischeri Cultures, Preparation of Juvenile E. scolopes, Inoculation Phase, Measurement of Bioluminescence, Euthanasia and Storage of Animals, Scoring of Bioluminescence

Preparation of *V. fischeri* Cultures

1 For each strain of interest, initiate a starter culture by inoculating 3 mL LBS with an isolated colony. Incubate starter cultures Overnight (~16 h) at 28 °C shaking at 200 rpm.

2 Measure the OD_{600} of each starter culture. In a microfuge tube, normalize each starter culture by diluting it to an OD_{600} of 1.0 in fresh LBS to a final volume of 1.0 mL. Vortex briefly.

3 Initiate an intermediate culture by inoculating 3 mL LBS in a fresh culture tube with 30 µL of the normalized cell suspension. Incubate at 28 °C shaking at 200 rpm.

Selection and Preparation of Juvenile *E. scolopes*

4 Using transfer pipet, collect freshly hatched juvenile squid into tumblers containing 100 mL FSSW, with no more than 50 squid/tumbler.

5 Prepare a new tumbler with 50 mL FSSW for each group.

6 Transfer animals from the 100 mL FSSW tumblers to the new tumblers individually .

Note

To minimize bias, add an animal to the tumbler of a different group with each transfer.

Preparation of Inoculums

7 For each strain, when the turbidity of culture is OD_{600} = 0.8-1.0, transfer culture volume equivalent to 1 mL of OD_{600} = 1.0 to a microfuge tube.

8 Concentrate cells by centrifugation.

8.1 Concentrate cells by centrifugation at 5000 x g, 00:02:30. Then, remove 0.9 mL supernatant, add 0.9 mL FSSW, and resuspend the pellet. (1/2)

2m 30s

8.2 Concentrate cells by centrifugation at 5000 x g, 00:02:30. Then, remove 0.9 mL supernatant, add 0.9 mL FSSW, and resuspend the pellet. (1/2)

2m 30s

9 Prepare a serial dilution by transferring 100 µL of the cell suspension described in Step 8 into 0.9 mL FSSW in a microfuge tube (10-1 dilution). Then, continue ten-fold dilutions until the desired dilution range has been achieved.

Note

Note that three-fold dilutions can be used instead for greater resolution.

10 Prepare a control for an apo-symbiotic group by transferring 1 mL FSSW to a microfuge tube.

11 For each group, transfer 100 µL from the corresponding microfuge tube into a 50-mL conical tube containing 50 mL FSSW and invert several times to mix.

Inoculation Phase

12 To initiate the inoculation phase, pour the cell suspension into the corresponding tumbler to bring the total volume to 100 mL. Repeat for the control described in Step 10.

13 Sample tumblers by plating 100 µL onto solid LBS medium in triplicate and incubate the plates at 28 °C Overnight.

Note

Note that for high inoculum levels, a dilution may be necessary to obtain countable CFUs. For low inoculum levels, it may be preferable to use the known dilution factor from more concentrated inoculums to estimate the corresponding abundance of *V. fischeri*.

14 After 3.5 hours, wash the animals by serially transferring them as a group into a tumbler containing 100 mL FSSW twice, with 00:05:00 between transfers. 5m

15 Transfer animals into vials containing 4 mL FSSW, with one animal per vial.

16 Store animals in a room that has a 12-h day/12-h night light cycle.

Measurement of Bioluminescence

17 After 16-18 h, transfer animals to clean vials containing 4 mL FSSW.

18 Using a luminometer, measure the luminescence emitted by each sample.

Euthanasia and Storage of Animals

19 To initiate the anesthesia step, transfer each animal with seawater (total volume of 0.5 mL) to a microfuge tube and place On ice.

20 After 00:05:00, add 0.5 mL cold 6% ethanol/FSSW to each microfuge tube and keep On ice. 5m

21 After 00:15:00, remove the liquid volume from the tube and store the anesthetized animal at -80 °C, thereby completing euthanasia. 15m

Scoring of Bioluminescence

22 Use the luminescence measurements of the apo-symbiotic group to determine the 99.9th percentile, above which animals are considered to be bioluminescent.

23 Score each animal as symbiotic or non-symbiotic by comparing the corresponding luminescence measurement with the bioluminescence cutoff defined in Step 22.

Determining Inoculum Levels

24 Count CFU on the inoculum plates generated in Step 13. Also verify that no CFU are present on the apo-symbiotic control plates.

25 Calculate the concentration of CFUs in each inoculum cell suspension described in Step 9 by dividing the CFU counts by the volume plated (in mL) and multiplying by the dilution factor, if any.

Calculation of SD_{50}

26 For each strain, generate a table with the number of symbiotic and non-symbiotic animals at each inoculum concentration, with rows arranged in order of highest to lowest concentration.

27 Prepare two additional columns containing adjusted counts for

1. animals that could be assumed to be symbiotic at higher inoculums and
2. animals that could be assumed to be non-symbiotic at lower inoculums.

28 Calculate the adjusted percent of symbiotic animals at each inoculum by dividing the adjusted counts of symbiotic animals by the total adjusted animal counts in the corresponding row.

29 Calculate the SD_{50} using the equation:

SD_{50} = 10^[log(DF^X) + log(c)], where

- X = [(50%-a)/(b-a)] and
- a = the adjusted percent symbiotic below 50% closest to 50%.
- b = the adjusted percent symbiotic above 50% closest to 50%.
- c = the inoculum concentration of the adjusted percent colonized below 50% closest to 50%.
- DF = the dilution factor or fold-change difference between groups in the experiment.
